# Endocrine Determinants of Changes in Insulin Sensitivity and Insulin Secretion during a Weight Cycle in Healthy Men

**DOI:** 10.1371/journal.pone.0117865

**Published:** 2015-02-27

**Authors:** Judith Karschin, Merit Lagerpusch, Janna Enderle, Ben Eggeling, Manfred J. Müller, Anja Bosy-Westphal

**Affiliations:** 1 Institute of Nutritional Medicine, University of Hohenheim, Stuttgart, Germany; 2 Institute of Human Nutrition and Food Science, Christian-Albrechts University, Kiel, Germany; Weill Cornell Medical College Qatar, QATAR

## Abstract

**Objective:**

Changes in insulin sensitivity (IS) and insulin secretion occur with perturbations in energy balance and glycemic load (GL) of the diet that may precede the development of insulin resistance and hyperinsulinemia. Determinants of changes in IS and insulin secretion with weight cycling in non-obese healthy subjects remain unclear.

**Methods:**

In a 6wk controlled 2-stage randomized dietary intervention 32 healthy men (26±4y, BMI: 24±2kg/m^2^) followed 1wk of overfeeding (OF), 3wks of caloric restriction (CR) containing either 50% or 65% carbohydrate (CHO) and 2wks of refeeding (RF) with the same amount of CHO but either low or high glycaemic index at ±50% energy requirement. Measures of IS (basal: HOMA-index, postprandial: Matsuda-ISI), insulin secretion (early: Stumvoll-index, total: tAUC-insulin/tAUC-glucose) and potential endocrine determinants (ghrelin, leptin, adiponectin, thyroid hormone levels, 24h-urinary catecholamine excretion) were assessed.

**Results:**

IS improved and insulin secretion decreased due to CR and normalized upon RF. Weight loss-induced improvements in basal and postprandial IS were associated with decreases in leptin and increases in ghrelin levels, respectively (r = 0.36 and r = 0.62, p<0.05). Weight regain-induced decrease in postprandial IS correlated with increases in adiponectin, fT3, TSH, GL of the diet and a decrease in ghrelin levels (r-values between -0.40 and 0.83, p<0.05) whereas increases in early and total insulin secretion were associated with a decrease in leptin/adiponectin-ratio (r = -0.52 and r = -0.46, p<0.05) and a decrease in fT4 (r = -0.38, p<0.05 for total insulin secretion only). After controlling for GL associations between RF-induced decrease in postprandial IS and increases in fT3 and TSH levels were no longer significant.

**Conclusion:**

Weight cycling induced changes in IS and insulin secretion were associated with changes in all measured hormones, except for catecholamine excretion. While leptin, adiponectin and ghrelin seem to be the major endocrine determinants of IS, leptin/adiponectin-ratio and fT4 levels may impact changes in insulin secretion with weight cycling.

**Trial Registration:**

ClinicalTrials.gov NCT01737034

## Introduction

Insulin resistance and hyperinsulinemia are early symptoms of metabolic dysfunction that precede the onset of type 2 diabetes. Even short-term overfeeding with modest weight gain contributes to decreased insulin sensitivity and compensatory increased insulin secretion [1,2]. Weight loss counteracts the negative consequences of a positive energy balance [3,4]; however, losses are rarely maintained, leading to recurrent weight cycling that has been associated with worsened metabolic and cardiovascular outcomes [5–7].

In addition, high glycemic index and glycemic load diets were independently associated with an increased risk of type 2 diabetes [8]. Because a diet high in rapidly absorbed carbohydrates and low in cereal fiber augments postprandial glycemia and insulin secretion it may contribute to glucotoxicity as well as increased insulin demand and eventually the development of insulin resistance.

To understand the etiology of early changes in IS and insulin secretion with perturbations in energy balance it is important to investigate healthy non-obese subjects, because a chronic disturbance in energy balance may increase visceral and liver fat and thus lead to impaired hepatic insulin clearance [9], chronic low grade inflammation [10] and elevated FFA levels [11] associated with IR and pathological hyperinsulinemia. Early determinants of changes in insulin sensitivity and insulin secretion that precede the development of ectopic fat accumulation could thus facilitate the identification of novel strategies for prevention of type 2 diabetes.

Several appetite-regulating hormones that fluctuate in response to changes in energy balance are known to potentially influence insulin levels or insulin sensitivity (e.g. leptin, ghrelin, GLP-1). Activation of proopiomelanocortin neurons by leptin has been shown to enhance insulin secretion [12] and degree of insulin resistance and β-cell function are associated with leptin in patients with Type 2 diabetes [13]. It is suggested that ghrelin has central and peripheral effects on glucose regulation and insulin level in humans [14,15] and GLP-1 has been shown to increase insulin secretion in a glucose-dependent manner [16]. However, changes in IS and insulin secretion with overfeeding and caloric restriction were accompanied by an unchanged GLP-1 response to oral glucose [4]. Moreover adiponectin [3], thyroid hormones and catecholamine excretion [17] change with body weight and might impact associated changes in IS or insulin secretion [18–20].

Early determinants of changes in IS and insulin secretion with weight loss and regain using different glycemic loads of the diet remain unclear. In a controlled dietary intervention, we therefore investigated the associations between changes in basal and OGTT-derived IS or insulin secretion with levels of ghrelin, leptin, adiponectin, thyroid hormones and catecholamine excretion during a weight cycle in 32 healthy men.

## Subjects and Methods

This paper focuses on a secondary aim of a previously published trial investigating the effect of carbohydrate intake and glycemic index on resting energy expenditure and substrate oxidation [21]. The study protocol was approved by the ethical committee of the Medical Faculty of the Christian-Albrechts-University of Kiel in November 2010. All participants provided informed written consent before participation according to the Declaration of Helsinki. No changes were made to the registered protocol. CONSORT checklist is available as supporting information; see S1 CONSORT Checklist.

Subjects and methods of this study have been described in more detail elsewhere [21,22]. Recruitment started in February 2011 and follow-up was finished in September 2012. Recruitment and final sample size for the analysis was 32 healthy men aged 20–37 years who were recruited at the University of Kiel campus. All subjects completed a medical history and physical examination to assess health status. Subjects did not use any medication on a daily basis and had a normal ECG-recording. Exclusion criteria for enrollment included smoking, unstable weight (>2 kg over the past 12 months, self-reported), family history of type 2 diabetes, food allergies, special diets (e.g. vegetarian) or being an athlete (i.e. >2h exercise per week or participation in competitive sports).

**Fig 1:**
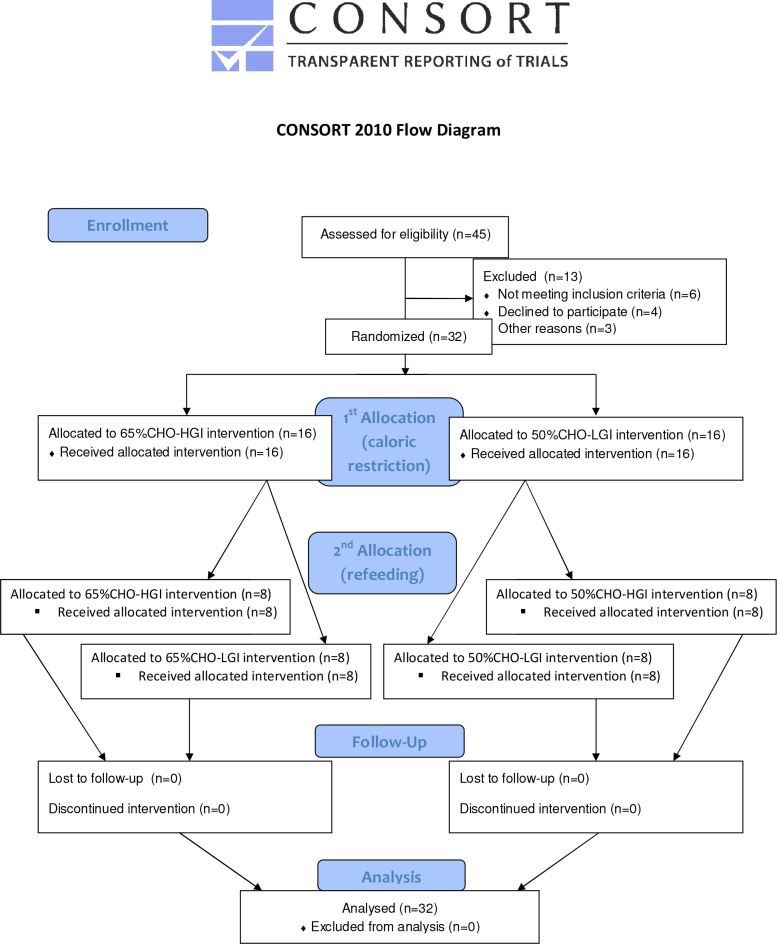
CONSORT flow chart, showing the passage of participants through the different stages of the present trial: enrollment, first allocation after OF to the 65%CHO and 50%CHO intervention and second allocation after CR to 65%CHO-HGI, 65%CHO-LGI, 50%CHO-HGI and 50%CHO-LGI intervention, follow-up, and analysis. OF, overfeeding; CR, caloric restriction; CHO: carbohydrate, HGI, high glyceamic index; LGI, low glyceamic index

CONSORT flow chart (Fig. 1) shows the passage of participants through the different stages of the present trial including enrollment, allocation to the interventions, follow-up, and analysis.

More detailed information of the study protocol is available as supporting information; see S1 Protocol.

### Study protocol

**Fig 2:**
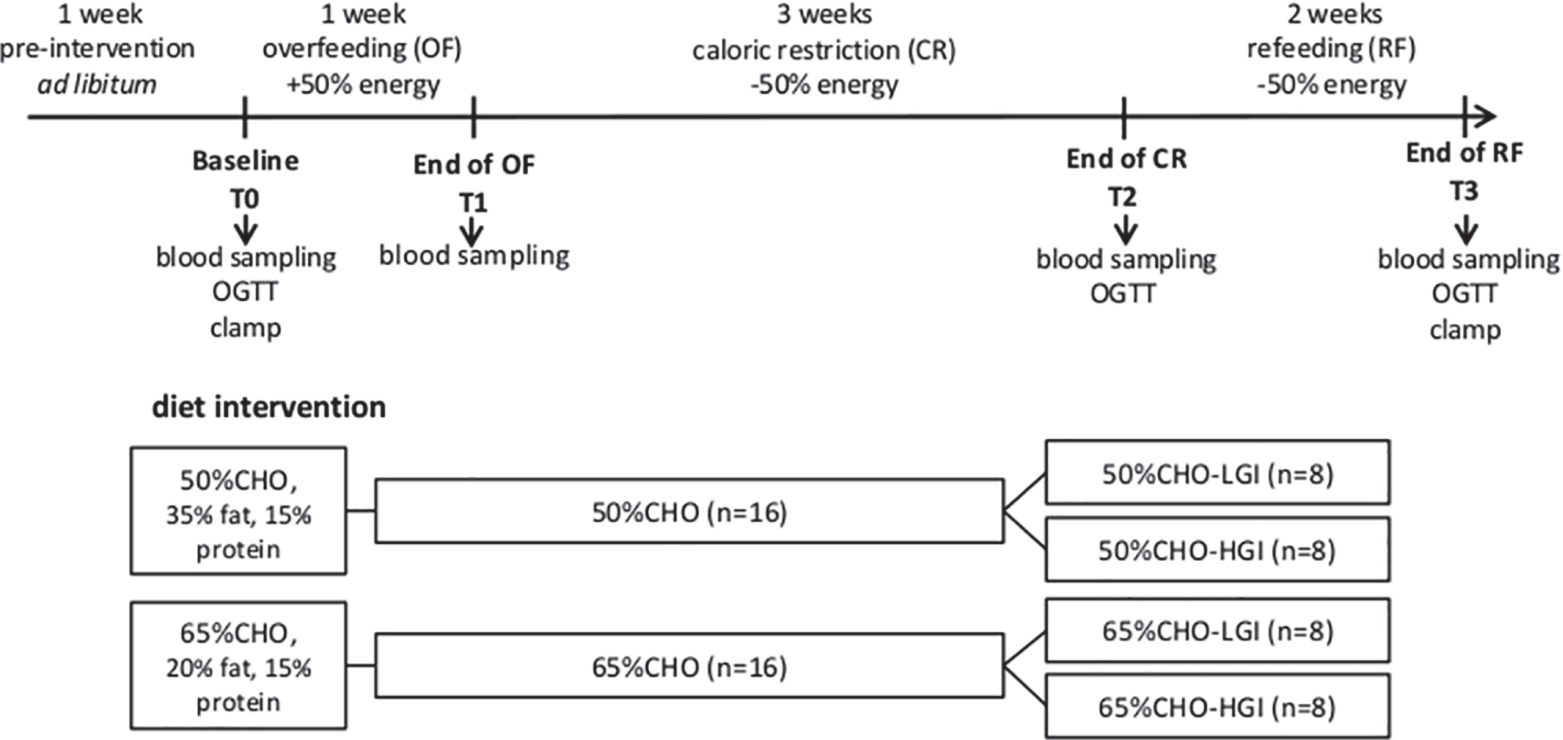
Schematic overview of the study protocol. OGTT, oral glucose tolerance test, CHO, carbohydrate; LGI, low glycaemic index; HGI, high glycaemic index

6-week strictly controlled dietary intervention was carried out at the Institute of Human Nutrition and Food Science at the Christian-Albrechts-University of Kiel. An outline of the study protocol is shown in Fig. 2. All subjects underwent an initial 1-week of overfeeding (OF, +50% of energy requirement), followed by 3-weeks of caloric restriction (CR, -50% of energy requirement) and subsequent 2-weeks of refeeding (RF, +50% of energy requirement). Energy requirement of each subject was defined by multiplying resting energy expenditure (measured by indirect calorimetry, Vmax Spectra 29n, SensorMedics, Viasys Healthcare, Bilthoven, Netherlands) by a sedentary physical activity level of 1.4 that resembles sedentary behavior [23]. Physical activity was checked throughout the study using Senswear activity monitors (SMT medical, Würzburg, Germany) (Mean values for PAL at baseline: 1.43 ±0.17; OF: 1.38 ±0.14; CR: 1.49 ±0.14; RF: 1.41 ±0.15).

Fasting blood samples were taken at baseline and at the end of each intervention period. An oral glucose tolerance test (OGTT) was conducted at baseline, as well as after CR and RF. Body weight was measured daily. Physical activity was monitored by step counters. Participants were informed that physical activity should not exceed 5,000 steps a day.

### Diet intervention

At the end of OF, subjects were randomized into a normal CHO intake (50% CHO, 35% fat, 15% protein) and a high CHO-intake group (65% CHO-HGI, 20% fat, 15% protein). After CR, both intervention-groups were further stratified into groups receiving a low glycaemic index (LGI) or a high glycaemic index (HGI) diet: 50%CHO-LGI, 50%CHO-HGI, 65%CHO-LGI and 65%CHO-HGI (Fig. 2). Details of the randomization process as well as basal characteristics of the study population stratified by intervention groups are given in our previous publication [22].

GL was calculated by multiplying the GI by the amount of CHO in grams provided by the food and dividing the total by 100 [24]. Mean values of dietary GL differed between the diet groups in both phases (CR: 50%CHO: 62 ±7; 65%CHO: 154 ±18, p<0.05; RF: 50%CHO-LGI: 213 ±16, 50%CHO-HGI: 356 ±57, 65%CHO-LGI: 259 ±33 and 65%CHO-HGI: 495 ±59, p<0.05).

### Weight status, body composition

Body weight was measured to the nearest 0.5 kg on a calibrated scale (seca 285, seca GmbH & Co.KG, Hamburg, Germany). Height was measured to the nearest 0.1 cm by stadiometer (seca 285, seca GmbH & Co.KG, Hamburg, Germany). Measurements were performed in underwear, without shoes after voiding. Fat-free mass and fat mass were measured by Quantitative Magnetic Resonance (QMR, EchoMRI-AH, Echo Medical Systems, Houston, Texas, USA).

### Blood sampling and analytical methods

Fasting blood samples were collected after an overnight fast (≥10h) at baseline and at the end of each intervention period. Glucose was measured using glucose oxidase method (BIOSEN C-Line, EKF-diagnostics, Texas, USA). Serum insulin was determined by electrochemiluminescence immunoassay (Elecsys, Roche diagnotics, Mannheim, Germany). Leptin, adiponectin, ghrelin and thyroid hormone levels (TSH, fT3, fT4) were measured by radioimmunoassay (RIA 125 Tube Kit, LINCO Research, St Charles, Missouri and Abott Diagnostics, Wiesbaden, Germany). Ghrelin secretion was shown to be inhibited by oral glucose administration [25], therefore ghrelin levels were measured at baseline and during OGTT. 24-h urinary catecholamine excretion (epinephrine and norepinephrine) was measured by HPLC according to Hollenbach et al, 1998 [26]. Because plasma norepinephrine and epinephrine have a circadian and ultradian rhythm 24h urinary catecholmine excretion was chosen as a more integrated measure of diurnal SNS activity.

### Oral glucose tolerance test

Participants underwent a standard OGTT (intake of 75 g glucose) at baseline as well as after CR and RF. Venous blood was sampled 0, 30, 90 and 120 minutes after glucose intake. Insulin, glucose and ghrelin responses were determined and calculated as incremental AUC (iAUC) and total AUC (tAUC) using the trapezoid rule [27].

### Hyperinsulinaemic-euglycaemic clamp

Whole-body IS at baseline and at the end of RF was assess by the hyperinsulinemic-euglycemic clamp technique according to DeFronzo et al [28], as previously described (1). *M*-value, expressed as mg/kg body weight/min, was determined during the last 20 min of the hyperinsulinemic glucose clamp as steady state glucose disposal rate calculated from the mean rate of exogenous glucose infusion corrected for glucose space.

### Calculations of fasting and postprandial IS and insulin secretion

Fasting IS was assessed using HOMA-index: fasting glucose (mmol/l) x fasting insulin (μU/l) /22.5 [29]. Postprandial IS was calculated by Matsuda whole-body IS Index (Matsuda-ISI): 10000/(√ (fasting glucose x fasting insulin) x (mean glucose x mean insulin during OGTT)) [30]. 1^st^-phase insulin secretion was estimated by Stumvoll-index: 1.283+1.829 x insulin_30min_—138.7 x glucose_30min_ + 3.772 x fasting insulin [31]. tAUC-insulin/tAUC-glucose was estimated to assess overall glucose-stimulated insulin secretion during OGTT [32].

### Statistical analysis

Data are presented as means ±SD. Analyses were performed using SPSS version 21.0 (SPSS Inc., Chicago, Illinois). Normal distribution was assessed by Kolmogorov-Smirnov test. Parameters liver fat and GL did not meet the criteria of normal distribution and were log transformed before correlation analyses. Repeated measures ANOVA was used to observe differences across the intervention periods followed by Bonferroni post hoc tests. Differences between diet groups (time x GI, time x CHO, time x GI x CHO, GI x CHO) were assessed by mixed-design ANOVA and Bonferroni post hoc tests. Associations between changes in fasting insulin, IS or insulin secretion and potential endocrine determinants were tested using Pearson’s correlation, whereas partial correlation analyses controlling for GL or liver fat were performed between RF-induced changes in Matsuda-ISI and changes in endocrine determinants. P-values <0.05 were considered statistically significant.

## Results

Baseline characteristics of the subjects and changes in body weight and fat mass during the intervention are summarized in Table 1. At baseline, seven subjects were overweight (BMI 25.6–29.3 kg/m^2^). Body weight and fat mass significantly increased due to OF (BW: 1.8 ±0.7 kg, p<0.001; FM: 0.8 ±0.6 kg, p<0.001), decreased with CR and increased again upon RF (all p<0.001). At the end of RF, mean body weight (p<0.05) and fat mass (p<0.001) were significantly lower compared to baseline. Changes in body weight and fat mass did not differ between diet groups except for the RF period. Weight regain was affected by CHO (<0.01) and GI x CHO interaction (p<0.05). Results of changes in body weight and fat mass by intervention groups on the same database are previously published [21]. In brief, weight regain was affected by CHO (<0.01) and GI x CHO interaction (p<0.05). Compared to the group with 50%CHO diet, subjects with 65%CHO diet gained more body weight (50%CHO: 3.0 ±1.2 kg; 65%CHO: 4.0 ±1.0 kg; p<0.05) particularly with HGI meals (50%CHO-LGI: 3.4 ±1.1 kg; 50%CHO-HGI: 2.5 ±1.1 kg; 65%CHO-LGI: 3.6 ±1.0 kg; 65%CHO-HGI: 4.4 ±0.6 kg; p<0.01) and fat mass regain was 60–70% higher in the 65%CHO-HGI group [21].

**Table 1:** Subject characteristics at baseline of the whole study population, and changes in body weight and fat mass during intervention periods, and changes of body weight and fat mass during CR and RF by intervention group.

	Baseline (T0)	OF (T1)	CR (T2)	RF (T3)	ΔCR (T2-T0)	ΔRF (T3-T2)
**age (years)**	25.5 ±3.9					
**height (cm)**	181.8 ±6.9					
**weight (kg)**	77.7 ±7.6	79.4 ±7.8^†††^	73.5 ±7.4	76.9 ±7.9	-4.2 ±0.9^†††^	3.5 ±1.2***
**FM (kg)**	13.8 ±5.1	14.6 ±5.3^†††^	12.0 ±5.0	13.2 ±5.0	-1.8 ±0.5^†††^	1.2 ±0.6***
**FM (%)**	17.8 ±5.9	18.4 ±5.8^†††^	16.3 ±6.0	17.2 ±5.7	-1.5 ±0.7^†††^	0.8 ±0.8***

Values are means ±SD, n = 32.

^†††^p<0.001 significantly different from baseline

***p<0.001 significantly different from previous period; Repeated measures ANOVA with Bonferroni adjustments

OF, overfeeding; CR, caloric restriction; RF, refeeding; FM, fat mass; CHO, carbohydrate; GI, glycemic index

At baseline, fasting insulin levels ranged from 1.3 to 22.4 mU/l and mean values of IS were in the normal range according to cut-off values by Radikova et al. (HOMA-IR <2.3 and Matsuda >5.0) [33], while three study participants had HOMA-IR values >2.3 and Matsuda indices <5.0 together with an increased FM (>23% FM). *M*-values were significantly associated with HOMA-index and Matsuda-ISI [22](r = -0.43 and r = 0.42, both p<0.05) at baseline as well as after RF with Matsuda-ISI (r = 0.55, p<0.05), while association with HOMA-IR tended to be associated (r = -0.34, p = 0.06).

### Changes in fasting insulin, IS, insulin secretion and potential endocrine determinants with overfeeding

Due to OF, fasting insulin levels and HOMA-index significantly increased (insulin: 4.05 ±4.83 mU/l, p<0.001; HOMA: 0.83 ±1.00, p<0.01) whereas glucose levels remained unchanged (Table 2). By contrast, no OF-induced changes in potential endocrine determinants were observed except for an increase in adiponectin (1.11 ±1.53 μg/ml, p<0.01) and a decrease in fT4 (-0.66 ±1.04 ng/l, p<0.01) (Table 3). No associations were observed between changes in fasting insulin levels or HOMA-index and potential endocrine determinants due to OF. Our discussion will therefore focus on the results of CR and RF.

**Table 2:** Changes in insulin secretion and IS due to overfeeding (OF) caloric restriction (CR) and refeeding (RF) (values are means ±SD).

	n	baseline (T0)	OF (T1)	CR (T2)	RF (T3)	∆CR (T2-T0)	∆RF (T3-T2)
**insulin secretion**							
insulin-tAUC/glucose-tAUC	30	9.43 ±5.68		7.77 ±4.14	10.25 ±7.25	-1.55 ±2.97^†^	2.34 ±4.22*
Stumvoll-index	30	1642 ±846		1300 ±512	1914 ±1114	-321 ±555^††^	576 ±840**
**fasting IS**							
insulin (mU/l)	32	7.94 ±4.15	11.99 ±6.26^†††^	5.59 ±3.69	8.13 ±4.36	-2.36 ±2.86^†††^	2.54 ±2.27***
glucose (mmol/l)	32	4.27 ±0.26	4.26 ±0.30	4.00 ±0.29	3.97 ±0.29	-0.27 ±0.34^†††^	-0.02 ±0.33
HOMA-index	32	1.48 ±1.10	2.39 ±1.27^††^	0.84 ±0.44	1.43 ±0.74	-0.63 ±0.93^††^	0.58 ±0.56***
**OGTT-derived IS**							
insulin-iAUC (mU/l per 2h)	30	104.2 ±50.0		98.0 ±50.1	116.6 ±89.5	-6.2 ±35.9	18.7 ±56.0
glucose-iAUC (mmol/l per 2h)	30	2.24 ±1.72		3.30 ±1.54	2.38 ±1.21	1.07 ±2.06^†^	-0.92 ±1.65*
Matsuda-ISI	30	9.17 ±4.44		12.13 ±6.37	8.45 ±3.46	2.95 ±3.23^†††^	-3.67 ±4.44***
							**∆RF-baseline (T3-T0)**
*M*-value (mg/kg/min)	31	9.0 ±2.9			8.7 ±2.2		-0.3 ±2.5

^†^p<0.05

^††^p<0.01

^†††^p<0.001 significantly different from baseline

*p<0.05

**p<0.01

***p<0.001 significantly different from previous period; Repeated measures ANOVA with Bonferroni adjustments

IS, insulin sensitivity; HOMA-index, homeostasis model assessment of insulin resistance; OGTT, oral glucose tolerance test; iAUC, incremental area under the curve; tAUC, total area under the curve; Matsuda-ISI, Mastsuda insulin sensitivity index

**Table 3:** Changes in endocrine parameters due to overfeeding (OF) caloric restriction (CR) and refeeding (RF) (values are means ±SD).

		Baseline (T0)	OF (T1)	CR (T2)	RF (T3)	ΔCR (T2-T0)	ΔRF (T3-T2)
**adipokines**							
leptin (ng/ml)	32	4.07 ±2.96	4.04 ±2.75	2.24 ±1.82	4.03 ±2.58	-1.83 ±2.40^†††^	1.79 ±1.47***
leptin/FM	32	0.27 ±0.12	0.26 ±0.10	0.18 ±0.11	0.29 ±0.11	-0.09 ±0.17^†^	0.11 ±0.11***
adiponectin (μg/ml)	32	8.16 ±3.20	9.26 ±3.07^††^	4.71 ±1.90	9.70 ±3.23	-3.45 ±2.18^†††^	4.99 ±2.89***
leptin/adiponectin	32	0.63 ±0.63	0.54 ±0.58	0.59 ±0.62	0.50 ±0.43	-0.04 ±0.51	-0.10 ±0.39
							
ghrelin (pg/l)	16	803 ±298	683 ±212	945 ±451	683 ±165	144 ±246	-262 ±356*
ghrelin-iAUC (pg/l per 2h)	16			-208 ±216	-91 ±113		117 ±206*
**thyroid hormones**							
fT3 (ng/l)	32	3.07 ±0.36	3.00 ±0.40	2.80 ±0.40	3.18 ±0.41	-0.27 ±0.42^††^	0.38 ±0.35***
fT4 (ng/l)	32	9.97 ±1.24	9.31 ±1.37^††^	10.42 ±1.17	9.04 ±1.05	0.45 ±1.09	-1.38 ±1.04***
TSH (mU/l)	32	2.07 ±1.06	1.86 ±0.90	1.51 ±0.74	2.21 ±0.94	-0.55 ±0.68^†††^	0.70 ±0.46***
**catecholamines**							
epinephrine (IU)	32	4.65 ±2.30	5.25 ±2.71	5.11 ±2.38	5.78 ±2.85	0.46 ±2.35	0.68 ±2.72
norepinephrine (IU)	32	40.5 ±12.3	38.8 ±8.5	26.5 ±7.9	38.2 ±9.9	-13.2 ±10.55^†††^	11. 6 ±7.8***

^†^p<0.05

^††^p<0.01

^†††^p<0.001 significantly different from baseline

*p<0.05

**p<0.01

***p<0.001 significantly different from previous period; Repeated measures ANOVA with Bonferroni adjustments

*OGTT*, oral glucose tolerance test; iAUC, incremental area under the curve

### Impact of the diet groups on changes in IS, insulin secretion and potential endocrine determinants

No time x CHO x GI interaction in changes of IS, insulin secretion or potential endocrine determinants between the diet groups were observed, except for a lower RF-induced reduction in Matsuda-ISI in the LGI groups compared to the HGI groups (LGI: -1.45 ±2.43; HGI: -5.90 ±4.92, p<0.05). Concomitantly, the increase in adiponectin with RF was higher in the HGI compared with the LGI group (HGI: 6.1 ±3.1 μg/ml; LGI: 3.9 ±2.3 μg/ml, p<0.05). GL was inversely associated with RF-induced decrease in Matsuda-ISI (r = -0.51; p<0.01).

### Changes in fasting insulin, IS, insulin secretion and potential endocrine determinants with CR and RF

**Fig 3:**
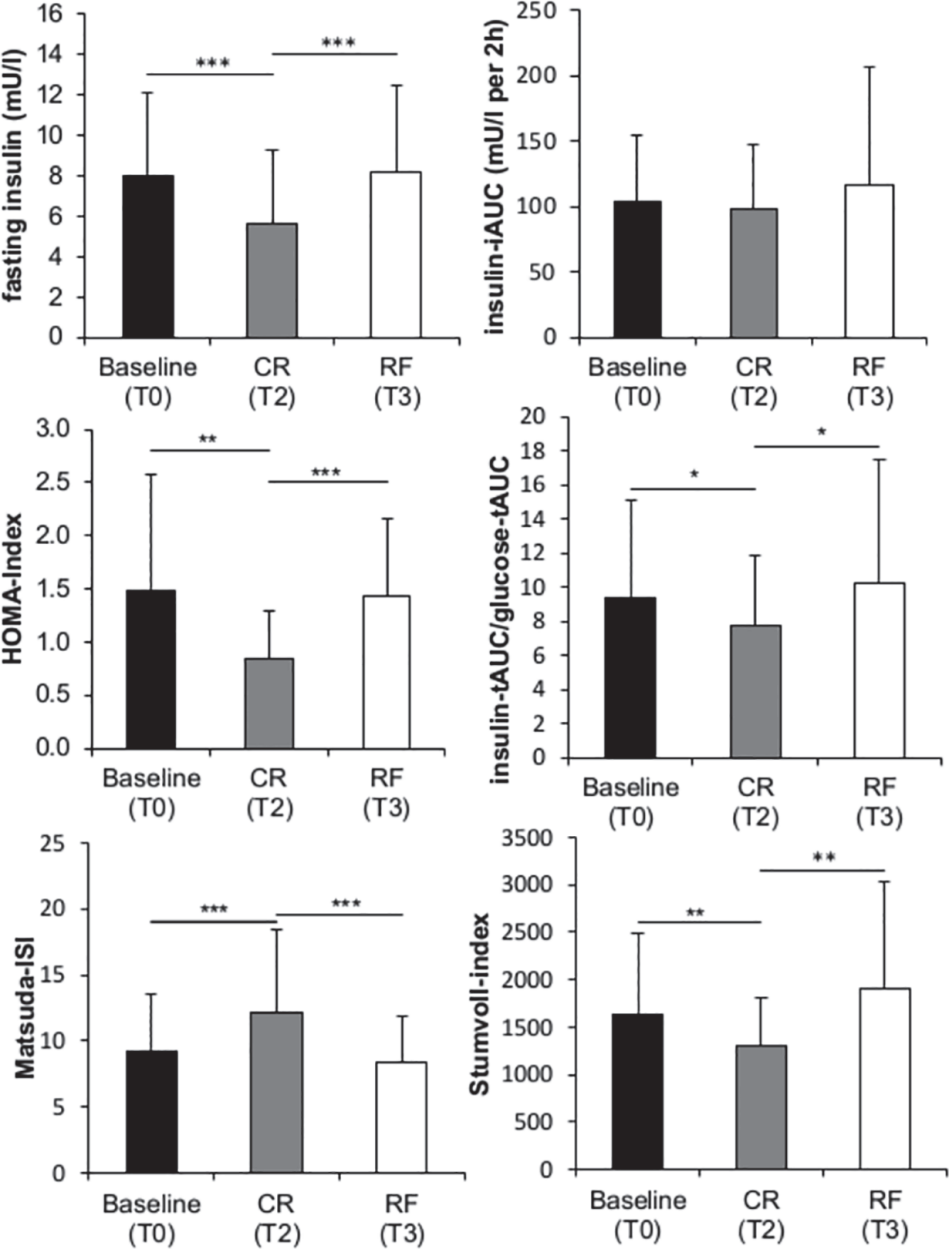
Comparison between fasting insulin level, IS (HOMA-index and Matsuda-ISI) as well as insulin secretion (insulin-iAUC, insulin-tAUC/glucose-tAUC, Stumvoll-index) at baseline (T0), after caloric restriction (CR, T2) and refeeding (RF, T3). *p<0.05, **p<0.01, ***p<0.001; Repeated measures ANOVA with Bonferroni adjustments. HOMA, homeostasis model assessment; ISI, insulin sensitivity index, iAUC, incremental area under the curve; tAUC, total area under the curve

Hyperinsulinaemic euglycemic clamp (*M*-value) revealed normal insulin sensitivity at baseline for all participants that remained unchanged after the weight cycle (Table 2). As shown in Fig. 3, due to CR, IS (HOMA-index, Matsuda-ISI) improved and fasting insulin as well as insulin secretion (insulin-tAUC/glucose-tAUC and Stumvoll-Index) decreased. All parameters normalized again upon RF (Fig. 3; Table 2). Fasting glucose levels decreased due to CR and remained unchanged upon RF (Table 2). Levels of leptin, leptin/FM, adiponectin, fT3, TSH as well as norepinephrine excretion significantly decreased with CR (Table 3). All parameters inversely changed upon RF. Fasting and postprandial ghrelin (ghrelin-iAUC) improved with RF. Whereas fT4 decreased with RF, leptin/adiponectin-ratio and epinephrine excretion remained unchanged during all intervention periods.

### Associations between changes in IS or insulin secretion and potential endocrine determinants with CR and RF

Improved IS due to CR correlated with the decrease in leptin (r = 0.36, p<0.01, for HOMA-index only) and the increase in ghrelin (r = 0.62, p<0.01, for Matsuda-ISI only). The decrease in postprandial IS upon RF was associated with the decrease in ghrelin (r = 0.83, r<0.001) as well as the increases in adiponectin (r = -0.38, p<0.05), fT3 (r = -0.40, r<0.05) and TSH (r = -0.38, r<0.05) levels. However, partial correlation analysis controlling for GL revealed that associations between the RF-induced decrease in Matsuda-ISI and increases in fT3 and TSH levels were no longer significant (fT3: r = -0.24; TSH: r = -0.32, both p>0.05).

The increase in insulin secretion upon RF (Stumvoll-index, insulin-tAUC/glucose-tAUC) was inversely associated with leptin/adiponectin-ratio (Stumvoll-index: r = -0.52; insulin-tAUC/glucose-tAUC: r = -0.46; both p<0.01) and fT4 levels (insulin-tAUC/glucose-tAUC: r = -0.38, p<0.05).

The decrease in fT3 and the increase in fT4 levels due to CR as well as inverse changes in these parameters with RF (Table 3) were positively associated with each other (CR: r = 0.39, p<0.05; RF: r = 0.39, p<0.05). Increases in fT3 and TSH levels upon RF were also correlated (r = 0.44, p<0.05).

Pearson's correlation coefficients (r) showing main interactions between changes in IS or insulin secretion and potential endocrine determinants are summarized in S1 Table.

## Discussion

In line with our hypothesis, weight loss and regain led to changes in IS and insulin secretion that were partly explained by concomitant changes in leptin, adiponectin, leptin/adiponectin-ratio, ghrelin, fT3 and fT4 levels, while the associations were not affected by different diet groups. Although the observed correlations need not necessarily be causal, our findings are supported by evidence from mechanistic, *in vitro*, animal or patient studies discussed below.

### Impact of leptin or leptin/adiponectin-ratio on IS or insulin secretion

The association between the decrease in leptin and the improvement in HOMA-index with CR (see results) was also shown with weight loss in obese patients [34]. Our results therefore indicate that even in mostly lean or slightly overweight subjects a diet-induced decrease in leptin is associated with improved insulin sensitivity. There is evidence for an adipoinsular axis in which insulin stimulates leptin production in adipocytes and leptin inhibits the production of insulin in human beta-cells [35]. We did however not find an inhibitory effect of leptin on insulin secretion during the weight cycle.

The ratio of leptin to adiponectin has been repeatedly suggested as an index of IS [36–38] whereas in our study we could not confirm an association between changes in leptin/adiponectin-ratio and IS. Adiponectin may however respond differently to caloric restriction in healthy non-obese insulin sensitive subjects compared to obese patients. While we found a decrease in adiponectin with a negative energy balance (Table 3) an increase in adiponectin with weight loss was observed in obese patients who also had lower baseline adiponectin levels [39]. Whereas the increase in adiponectin with weight loss in obese patients was associated with improved IS [40] in our study the decrease in adiponectin with caloric restriction was accompanied by an improvement in IS and vice versa upon RF (Table 2 and 3). The proposed insulin sensitizing effect of adiponectin [41] therefore needs further investigation. The observed changes in adiponectin are in line with other studies in lean healthy men [42,43] that imply that the coincidence of changes in adiponectin and IS may not be causal in non-obese healthy subjects.

Although it was shown that adiponectin increased insulin secretion in human pancreatic β-cells [44] we found no association between changes in adiponectin and increased insulin secretion during refeeding. This may be due to the observation that adiponectin exerts its effect on insulin secretion by the activation of lipid oxidation [44] that is suppressed with hypercaloric refeeding.

### Impact of ghrelin on IS or insulin secretion

Increases in ghrelin levels with caloric restriction were associated with an improvement in Matsuda-ISI. Likewise reduced ghrelin levels with RF were associated with a decrease in Matsuda-ISI. These findings are consistent with cross-sectional data from two studies that found circulating ghrelin levels to be associated with lower insulin resistance (assessed by HOMA-index) [45,46]. In line with a potential positive effect of ghrelin on glucose homeostasis, fasting ghrelin is lower in obese insulin resistant adolescents with polycystic ovary syndrome [45] and type 2 diabetic patients [47]. The insulin sensitizing effect of ghrelin remains controversial because IS measured by the gold standard euglycemic hyperinsulinemic clamp, was not associated with plasma ghrelin in a Swedish population of healthy men [48]. Moreover, an acute administration of exogenous ghrelin has been found to decrease glucose stimulated insulin secretion in healthy humans [14,49,50], an effect that could be mediated by increased AMPK phosphorylation and UCP2 mRNA expression [51]. However, we found no association between changes in ghrelin levels and insulin secretion in response to weight loss or weight regain.

### Impact of thyroid hormones on IS or insulin secretion

In contrast to normal thyroid regulation TSH and fT3 did not change in opposite directions but showed a joint decrease due to CR and increase upon RF (with inverse changes in fT4 levels), respectively (Table 3). These findings are in line with the observation of low levels of fT3 and TSH in underweight patients [52,53] and elevated levels of both hormones in obese patients [54,55]. We found that the changes in fT3 and fT4 due to CR and RF were correlated (see results). This may be explained by impaired (CR) and increased (RF) extrathyroidal monodeiodination of T4 into T3 that was observed in underweight and overweight subjects, respectively [56]. Low fT3 in underweight and high levels in overweight subjects have been suggested to reflect metabolic adaptation to the altered nutritional status to spare energy consumption in starvation or increase energy expenditure in overfeeding [57].Our data support this hypothesis and show that even short-term perturbations in energy balance are associated with adaptations of thyroid hormone levels.

Increases in fT3 and TSH with RF were associated with a decrease in postprandial IS assessed by Matsuda-ISI (Table 3). Similar findings have also been shown in subjects with elevated TSH or fT3 levels [19]. Nevertheless, associations between thyroid hormones and IS remain controversial since not only hyperthyroidism but also hypothyroidism was associated with insulin resistance [57]. Explanations for the underlying mechanisms mostly refer to the insulin antagonistic effect of thyroid hormones in the liver or insulin agonistic effects in the muscle, found in animal models [58]. However, these results were obtained in hyper- or hypothyroidism and cannot be compared to our results in healthy humans with changes in thyroid hormones in a normal euthyroid range. Ortega et al. found a positive association between insulin secretion and fT3 in euthyroid humans [20]. Although we found an inverse correlation between refeeding-induced changes in fT4 and insulin secretion (assessed by tAUC-insulin/tAUC-glucose) a decrease in fT4 with refeeding correlated with an increase in fT3 levels (see results) and imply an increased T4-T3 conversion that might facilitate a higher insulin secretion.

Associations between changes in Matsuda-ISI and thyroid hormones with RF were no longer significant using partial correlation analysis adjusting for GL (see results). This suggests that the decrease in IS with refeeding is not explained by an increase in fT3 but is due to the increase in GL of the diet that has been shown to contribute to increased fT4 to fT3 conversion in rats [59]. In line with this interpretation, it was shown that fT3 levels increased 21% during three weeks of carbohydrate overfeeding [60].

### Impact of catecholamine excretion on IS or insulin secretion

Norepinephrine excretion decreased with CR and increased again after RF (with a concomittant increase in epinephrine). No associations were observed between changes in catecholamine excretion and changes in IS or insulin secretion. It has however long been known from isolated beta cells in a rat model that epinephrine and norepinephrine inhibit insulin secretion [61–63]. It was also shown, that euglycaemic hyperinsulinemia during a clamp leads to an increase in norepinephrine release indicating an increase in SNS activity [64] and thus contributes to impaired IS [65]. Since it is assumed that only chronic or sustained SNS activation contribute to insulin resistance [66], short-term changes in SNS-activity during two weeks of hypercaloric refeeding may not be long enough to impair IS.

### Strengths and limitations of the study

A major advantage of our study is the controlled dietary intervention in healthy subjects that allows an intraindividual comparison of early determinants of changes in insulin sensitivity or insulin secretion with weight loss and regain.

There are also some limitations that need to be discussed. (i) Deterioration in IS and secretion with RF may not only be explained by endocrine determinants but may also be due to an increase in ectopic lipid storage. In a previous publication on the same database, liver fat increased with RF above baseline values [22]. We now observed that liver fat after RF was positively associated with RF-induced changes in HOMA-IR and insulin secretion (HOMA-IR: r = 0.55; Stumvoll-index: r = 0.65 and insulin-tAUC/glucose-tAUC: r = 0.70, all p<0.05). Using partial correlation adjusting for liver fat, associations between RF-induced changes in Matsuda-ISI and thyroid hormones or insulin secretion and thyroid hormones as well as leptin/adiponectin-ratio were no longer significant (data not shown). (ii) Indirect methods were used to assess IS (HOMA-index, Matsuda-ISI). Although surrogate measures of insulin sensitivity are preferred because they are less expensive, invasive and challenging to perform they should be used with caution in longitudinal study designs because they showed less correlation with reference methods of IS in a an intervention study [67]. (iii) Although we did not measure GLP-1, a recent study showed no changes in basal or postprandial GLP-1 after overfeeding for 35 days and subsequent caloric restriction in healthy men [4]. By contrast, undercarboxylated osteocalcin and cortisol may be better determinants of changes in IS and insulin secretion with weight loss and regain. Of notice, recent studies showed a correlation between serum/plasma osteocalcin and insulin secretion in lean subjects [68] and the weight-loss induced increase in osteocalcin was associated with improved glucose homeostasis [69]. In obese children, weight-loss induced reduction in cortisol correlates with improvement in IS. Serum testosterone may also be an important determinant of changes in IS during weight cycling. Short-term starvation decreased serum testosterone levels by approximately 40% in lean healthy men [70] and low serum testosterone levels have been shown to be associated with insulin resistance [71]. Further studies focusing insulin secretion should also address arterial natriuretic peptide (ANP) since recent studies found increased insulin levels during ANP infusion in human subjects [72].(iv) Because our intervention involved changes in energy balance as well as diet composition this may limit the power of our analysis. As a measure of effect size, i.e. the practical meaningfulness of main effects and diet interactions, we used SPSS-derived partial eta squared, to describe effect sizes in our ANOVA. Mean partial eta squared for all main effects (caloric restriction and refeeding per se) was 0.52, and partial eta squared for all time x diet interactions (glycemic index and carbohydrate content) averaged 0.07. According to Cohen (1988, cited by Westermann R, 2000 [73]) these partial eta squared values are of large (>0.1) and medium size (0.06–0.1), respectively. This shows that the energy deficit or surplus alone has a much stronger impact on changes in outcome variables and may override some effects of the diet composition.

### Conclusion

Even in non-obese healthy men changes in leptin contribute to changes in IS after weight loss. We could not confirm that leptin/adiponectin-ratio is an important determinant of IS during weight loss or regain. By contrast, it is suggested that adiponectin and ghrelin affect IS during weight cycling. Changes in thyroid hormones might reflect early adaptations in energy balance. While leptin, ghrelin and adiponectin seem to be the major endocrine determinants of changes in IS, leptin/adiponectin-ratio and fT4 levels may impact changes in insulin secretion with weight cycling. Catecholamine excretion does not seem to be involved in early changes in IS or insulin secretion with short-term perturbations in energy balance.

## Supporting Information

S1 CONSORT Checklist CONSORT checklist. (DOC)

S1 Protocol Outline of the study protocol. (DOC)

S1 Table Pearson correlation coefficients between changes in IS or insulin secretion and endocrine determinants due to caloric restriction (CR) and refeeding (RF). (DOC)
