# Supplementary material for: Endocrine Determinants of Changes in Insulin Sensitivity and Insulin Secretion during a Weight Cycle in Healthy Men
Source: PLoS One. 2015 Feb 27;10(2):e0117865. doi: 10.1371/journal.pone.0117865 (PMC4344201; doi:10.1371/journal.pone.0117865)
Supplement: S1 Protocol — (DOC) [file pone.0117865.s002.doc]

**Outline of the study protocol**

*Subject eligibility and recruitment*

Data collection is planned between February 2010 and September 2012. Recruitment and final sample size for the analysis should be 32 healthy men, 20-40 years old, who will be recruited at the University of Kiel campus. Since metabolic changes and dysregulation in energy balance is assumed in obese subjects, only healthy and normal weight subjects will be recruited. To assess eligibility of the subjects, they will complete a medical history and physical examination to assess health status. Exclusion criteria for enrollment include

- Smoking
- unstable weight (>2 kg over the past 12 months)
- chronic diseases
- regular use of medications
- family history of type 2 diabetes
- food allergies or special diets (e.g. vegetarian)
- being an athlete.

*Study protocol*

6-week strictly controlled dietary intervention will be carried out at the Institute of Human Nutrition and Food Science at the Christian-Albrechts-University of Kiel. An outline of the study protocol is shown in figure 1. Before the dietary intervention, there is a 1-week pre-intervention period, during which each subject will undergo a hyperinsulinemic euglycemic clamp and an oral glucose tolerance test (OGTT). Prior to the tests, resting energy expenditure (REE) will be measured to determine individual energy requirement during the intervention. Body weight and blood pressure will be measured daily throughout the 6-week dietary intervention.

All subjects will undergo an initial 1-week of overfeeding (OF, +50% of energy requirement), followed by 3-weeks of caloric restriction (CR, -50% of energy requirement) and subsequent 2-weeks of refeeding (RF, +50% of energy requirement). The initial overfeeding period is added to the study protocol to limit minimum body weight. Energy requirement of each subject will be calculated by multiplying resting energy expenditure (REE, measured by indirect calorimetry, Vmax Spectra 29n, SensorMedics®, Viasys Healthcare, Bilthoven, Netherlands) by a sedentary physical activity level of 1.4. Fasting blood samples will be taken at baseline and at the end of each intervention period. An OGTT will be conducted at baseline, as well as after CR and RF. Body weight will be measured daily. Physical activity will be monitored by step counters. Participants will be informed that physical activity should not exceed 5,000 steps a day.

Measurements of REE and OGTT as well as fasting blood sampling will be repeated at the end of caloric restriction and refeeding. Continuous interstitial glucose monitoring, glycemic index testing, and a second euglycemic clamp will be performed at the end of the refeeding period.


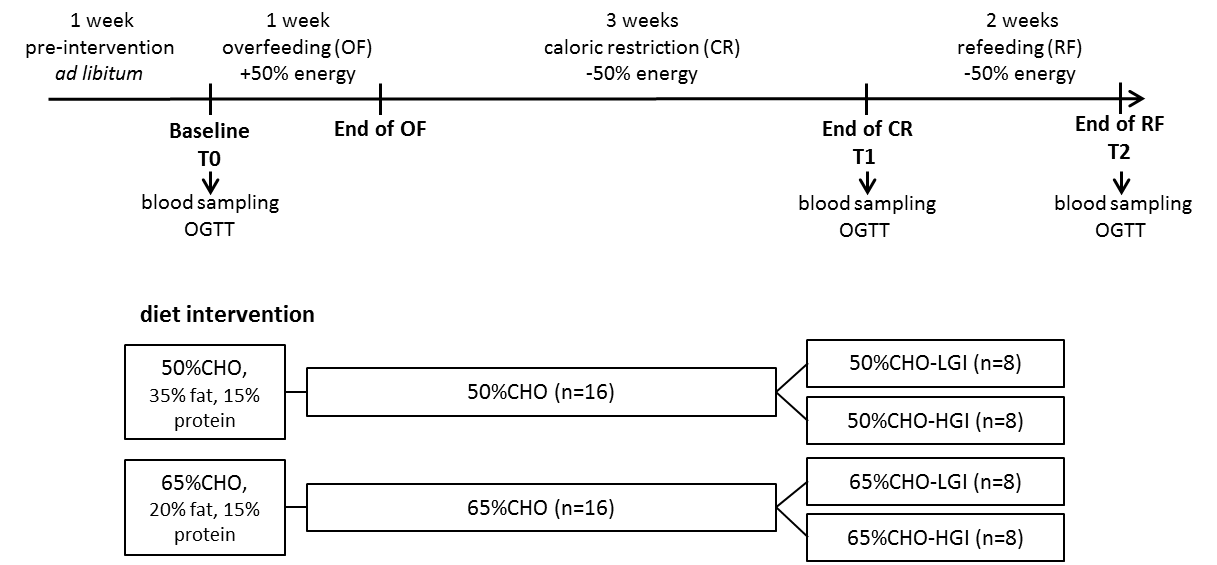


Figure 1: schematic overview of the study protocol; OGTT, oral glucose tolerance test; CHO, carbohydrate; HGI, high glycaemic index; LGI, low glycaemic index

Participants will arrive at the metabolic ward of the Institute of Human Nutrition and Food Science every morning at 8.30 a.m. after an overnight fast of ≥ 10 h. During the pre-intervention period, they are allowed to leave the institute after all measurements are completed. Throughout the intervention periods, subjects are requested to stay at the institute until dinner.

*Main aim*

Main aim of the study is to determine the impact of glycemic index and glycemic load on insulin sensitivity during a weight cycle in young healthy men. Additionally we would like to assess the effect of carbohydrate intake and glycemic index on resting energy expenditure and substrate oxidation during a strictly controlled diet with caloric restriction and refeeding.

*Secondary aim*

Secondary aim of the study is to determine endocrine determinants of insulin sensitivity and insulin secretion during caloric restriction and refeeding.

**Methods**

*Diet intervention*

At the end of OF, subjects will be randomized into a normal CHO-intake (50% CHO, 35% fat, 15% protein) and a high CHO-intake group (65% CHO, 20% fat, 15% protein). After CR, both intervention-groups will be further stratified into groups receiving a low glycaemic index (LGI) or a high glycaemic index (HGI) diet: 50%CHO‑LGI, 50%CHO‑HGI, 65%CHO‑LGI and 65%CHO‑HGI (figure 1).

All foods and beverages consumed will be provided during the intervention. Seven-day cycle menus are prepared by an experienced dietitian by using PRODI® software (Nutri-Science GmbH, Freiburg, Germany). GI values of individual foods are taken from published international tables [1]. The GI of the diets is calculated according to WHO/FAO guidelines [2] and averaged 34 units in the LGI and 58 units in the HGI diet, respectively. All diets consist of 50% of total daily energy as carbohydrate, 35% as fat, and 15% as protein. Refeeding diets are not matched for fiber content. Subjects will be instructed to eat all food provided and meal intake will be supervised by a skilled nutritionist. Participants will be allowed to drink water, decaffeinated coffee and tea ad libitum and fluid intake was recorded.

During the initial overfeeding period, subjects will receive a normal mixed-diet. During caloric restriction and LGI vs. HGI refeeding, 50% of the energy intake will be given as a liquid formula diet that is provided free of charge by the InsuLean company: During caloric restriction, the subjects will consume two liquid formula meals per day (InsuLean Vital Protein Creme Royal, InsuLean GmbH & Co. KG, Essen, Germany). During HGI refeeding, two liquid formula meals will be prepared with lactose-free milk (InsuLean Pur, InsuLean GmbH & Co. KG, Essen, Germany) and two servings of grape juice will be enriched with maltodextrin. During LGI refeeding, three liquid formula meals (InsuLean Vital Protein Creme Royal), will be prepared with soy-milk and enriched with lactose and sucrose.

An individual GI of the test meals will be calculated for each participant according to the following equation: glucose iAUC test meal / glucose iAUC OGTT * 100 [3].

The remaining 50% of kilocalories will be provided as HGI and LGI mixed-meals and snacks, respectively. Lower or higher GI versions of key “staple” carbohydrate-rich foods are incorporated into the diet (e.g. lower or higher GI breads, rice, pasta, potato products).

*Anthropometric measurements and body composition analysis*

Height will be measured to the nearest 0.1 cm using a stadiometer, with subjects not wearing shoes. Body weight will be measured to the nearest 0.05 kg on an electronic scale (seca 285, seca GmbH & Co KG, Hamburg, Germany) with subjects in underwear and after voiding. Fat mass and fat-free mass will be assessed by Quantitative Magnetic Resonance (QMR, EchoMRI-AH™, Echo Medical Systems, Houston, Texas, USA). Liver fat will be determined by MRI (Magnetom Avanto 1.5-T Siemens, Erlangen, Germany) along with the two-point Dixon method with a volume interpolated breath hold examination.

*Energy expenditure*

Respiratory exchange measurements will be performed by means of an open-circuit indirect calorimeter (ventilated hood system Vmax 29n, SensorMedics®; Sensor Medics 130 GmbH Höchberg, Germany). Oxygen consumption and carbon dioxide production will be continuously measured for ≥ 30 min and REE will be calculated from steady state intervals using a standard formula. Activity energy expenditure (AEE) will be assessed by the SenseWear Pro3 Armband (Body-Media Inc., Pittsburgh, PA) that will be continuously worn on the subjects´ dominant arm.

*Blood sampling and analytical methods*

Fasting blood samples will be collected after an overnight fast (≥10h) at baseline and at the end of each intervention period. Glucose will be measured using glucose oxidase method (BIOSEN C-Line, EKF-diagnostics, Texas, USA). Serum insulin will be determined by electrochemiluminescence immunoassay (Elecsys®, Roche diagnotics, Mannheim, Germany). Leptin, adiponectin, ghrelin and thyroid hormone levels (TSH, fT3, fT4) will measured by radioimmunoassay (RIA 125 Tube Kit, LINCO Research, St Charles, Missouri and Abott Diagnostics, Wiesbaden, Germany). 24-h urinary catecholamine excretion (epinephrine and norepinephrine) will measured by HPLC according to Hollenbach et al, 1998 [4].

*Oral glucose tolerance test*

Participants will undergo a standard OGTT (intake of 75 g glucose) at baseline as well as after CR and RF. Venous blood will be sampled 0, 30, 90 and 120 minutes after glucose intake. Insulin, glucose and ghrelin responses will determined and calculated as incremental AUC (iAUC) and total AUC (tAUC) using the trapezoid rule [5].

*Continuous glucose monitoring*

Subcutaneous interstitial fluid glucose levels will be measured by means of the FreeStyle Navigator® continuous glucose monitoring (CGM) device (Abbott Diabetes Care, Alameda, CA, USA) at the end of the refeeding period. A CGM monitor will be worn for 5 consecutive days, data will be downloaded and glucose profiles will be analyzed. Interstitial glucose iAUC during the day (8.00 -10.00 h), i.e. daytime glycemia, and tAUC during the night (0.00-7.00 h), i.e. nighttime glycemia, will be calculated using the trapezoidal rule [5].

*Hyperinsulinaemic-euglycaemic clamp*

Whole-body IS at baseline and at the end of RF will be assess by the hyperinsulinemic-euglycemic clamp technique according to DeFronzo et al [6]. *M*-value, expressed as mg/kg body weight/min, will be determined during the last 20 min of the hyperinsulinemic glucose clamp as steady state glucose disposal rate calculated from the mean rate of exogenous glucose infusion corrected for glucose space.

*Calculations of fasting and postprandial IS and insulin secretion*

Fasting IS will be assessed using HOMA-index: fasting glucose (mmol/l) x fasting insulin (µU/l) /22.5 [7]. Postprandial IS will be calculated by Matsuda whole-body IS Index (Matsuda-ISI): 10000/(√ (fasting glucose x fasting insulin) x (mean glucose x mean insulin during OGTT)) [8]. 1^st^-phase insulin secretion will be estimated by Stumvoll-index: 1.283+1.829 x insulin_30min_ – 138.7 x glucose_30min_ + 3.772 x fasting insulin [9]. tAUC-insulin/tAUC-glucose will be estimated to assess overall glucose-stimulated insulin secretion during OGTT [10].

*Statistical analysis*

Cross-sectional analysis between different diet groups will be performed using ANOVA (analysis of variance) after adjustment for co-variables. Longitudinal analysis between caloric restriction and refeeding will be assessed using repeated measures ANOVA. Unpaired t-test indicates that enrollment of 32 subjects (16 each diet group) will provide 75% power for the primary aim of impact of carbohydrate intake on insulin sensitivity with a 15% higher Matsuda-ISI after CR in the 50%CHO diet group compared to the 65%CHO diet group. Differences were considered significant if p ≤ 0.05.

1. Foster-Powell K, Holt SH, Brand-Miller JC (2002) International table of glycemic index and glycemic load values: 2002. Am J Clin Nutr 76: 5–56.

2. Carbohydrates in human nutrition. Report of a Joint FAO/WHO Expert Consultation. (1998). FAO Food Nutr Pap 66: 1–140..

3. Jenkins DJ, Wolever TM, Taylor RH, Barker H, Fielden H, et al. (1981) Glycemic index of foods: a physiological basis for carbohydrate exchange. Am J Clin Nutr 34: 362–366..

4. Hollenbach E, Schulz C, Lehnert H (1998) Rapid and sensitive determination of catecholamines and the metabolite 3-methoxy-4-hydroxyphen-ethyleneglycol using HPLC following novel extraction procedures. Life Sci 63: 737–750.

5. Matthews JN, Altman DG, Campbell MJ, Royston P (1990) Analysis of serial measurements in medical research. BMJ 300: 230–235.

6. DeFronzo R, Tobin J, Andres R (1979) Glucose clamp technique: a method for quantifying insulin secretion and resistance. Am J Physiol Endocrinol Metab 237: E214–223..

7. Matthews DR, Hosker JP, Rudenski AS, Naylor BA, Treacher DF, et al. (1985) Homeostasis model assessment: insulin resistance and beta-cell function from fasting plasma glucose and insulin concentrations in man. Diabetologia 28: 412–419.

8. Matsuda M, DeFronzo R (1999) Insulin sensitivity indices obtained from oral glucose tolerance testing: comparison with the euglycemic insulin clamp. Diabetes Care 22..

9. Stumvoll M, Mitrakou a, Pimenta W, Jenssen T, Yki-Järvinen H, et al. (2000) Use of the oral glucose tolerance test to assess insulin release and insulin sensitivity. Diabetes Care 23: 295–301.

10. Albareda M, Murugo M, Leiva A De, Corcoy R (2000) Assessment of insulin sensitivity and beta-cell function from measurements in the fasting state and during an oral glucose tolerance test: 1507–1511.
