# Supplementary material for: Endocrine Determinants of Changes in Insulin Sensitivity and Insulin Secretion during a Weight Cycle in Healthy Men
Source: PLoS One. 2015 Feb 27;10(2):e0117865. doi: 10.1371/journal.pone.0117865 (PMC4344201; doi:10.1371/journal.pone.0117865)
Supplement: S1 Table — (DOC) [file pone.0117865.s003.doc]

Table S1: Pearson correlation coefficients between changes in IS or insulin secretion and endocrine determinants due to caloric restriction (CR) and refeeding (RF)

|  | **Δ HOMA-IR** | | **Δ Matsuda-ISI** | |  | **Δ Stumvoll-Index** | | **Δ tAUC-insulin/tAUC-glucose** | |
| --- | --- | --- | --- | --- | --- | --- | --- | --- | --- |
|  | **CR** | **RF** | **CR** | **RF** |  | **CR** | **RF** | **CR** | **RF** |
| **Adipokines** |  |  |  |  |  |  |  |  |  |
| Δ leptin | **0.36*** | 0.28 | 0.16 | 0.14 |  | 0.12 | 0.03 | 0.12 | 0.21 |
| Δ adiponectin | -0.16 | -0.01 | -0.27 | **-0.38*** |  | -0.27 | 0.02 | -0.29 | 0.07 |
| Δ leptin/adiponectin | 0.04 | -0.06 | 0.21 | 0.05 |  | -0.19 | **-0.52**** | -0.13 | **-0.46**** |
|  |  |  |  |  |  |  |  |  |  |
| Δ ghrelin | 0.19 | -0.03 | **0.62*** | **0.83***** |  | -0.04 | -0.25 | -0.19 | -0.37 |
| **Thyroid hormones** |  |  |  |  |  |  |  |  |  |
| Δ fT3 | -0.15 | -0.06 | -0.17 | **-0.40*** |  | -0.23 | -0.02 | 0.03 | -0.07 |
| Δ fT4 | -0.02 | -0.30 | -0.18 | -0.21 |  | -0.12 | -0.28 | 0.02 | **-0.38*** |
| Δ TSH | 0.11 | 0.19 | 0.14 | **-0.38*** |  | -0.08 | 0.18 | 0.04 | 0.22 |

*p<0.05, **p<0.01, ***p<0.001

IS, insulin sensitivity; HOMA-index, homeostasis model assessment of insulin resistance; OGTT, oral glucose tolerance test; iAUC, incremental area under the curve; tAUC, total area under the curve; Matsuda-ISI, Mastsuda insulin sensitivity index
